# The endogenous hydrogen sulfide producing enzyme cystathionine-β synthase contributes to visceral hypersensitivity in a rat model of irritable bowel syndrome

**DOI:** 10.1186/1744-8069-5-44

**Published:** 2009-08-06

**Authors:** Guang-Yin Xu, John H Winston, Mohan Shenoy, Shufang Zhou, Jiande DZ Chen, Pankaj J Pasricha

**Affiliations:** 1 Division of Gastroenterology and Hepatology, Department of Internal Medicine, University of Texas Medical Branch, Galveston, Texas 77555-0655, USA; 2 Department of Gastroenterology and Hepatology, Stanford University School of Medicine, Stanford, California 94305-5187, USA

## Abstract

**Background:**

The pathogenesis of visceral hypersensitivity, a characteristic pathophysiological feature of irritable bowel syndrome (IBS), remains elusive. Recent studies suggest a role for hydrogen sulfide (H_2_S) in pain signaling but this has not been well studied in visceral models of hyperalgesia. We therefore determined the role for the endogenous H_2_S producing enzyme cystathionine-β-synthetase (CBS) in a validated rat model of IBS-like chronic visceral hyperalgesia (CVH). CVH was induced by colonic injection of 0.5% acetic acid (AA) in 10-day-old rats and experiments were performed at 8–10 weeks of age. Dorsal root ganglion (DRG) neurons innervating the colon were labeled by injection of DiI (1,1'-dioleyl-3,3,3',3-tetramethylindocarbocyanine methanesulfonate) into the colon wall.

**Results:**

In rat DRG, CBS-immunoreactivity was observed in approximately 85% of predominantly small- and medium-sized neurons. Colon specific DRG neurons revealed by retrograde labeling DiI were all CBS-positive. CBS-positive colon neurons co-expressed TRPV1 or P2X3 receptors. Western blotting analysis showed that CBS expression was significantly increased in colon DRGs 8 weeks after neonatal AA-treatment. Furthermore, the CBS inhibitor hydroxylamine markedly attenuated the abdominal withdrawal reflex scores in response to colorectal distention in rats with CVH. By contrast, the H_2_S donor NaHS significantly enhanced the frequency of action potentials of colon specific DRG neurons evoked by 2 times rheobase electrical stimulation.

**Conclusion:**

Our results suggest that upregulation of CBS expression in colonic DRG neurons and H_2_S signaling may play an important role in developing CVH, thus identifying a specific neurobiological target for the treatment of CVH in functional bowel syndromes.

## Background

Hydrogen sulfide (H_2_S), a gas synthesized by sulfate reducing colonic bacteria and the endogenous enzymes cystathionine-*β*-synthetase (CBS) and cystathionine-*γ*-lyase (CSE) [1-5], is increasingly recognized as a biologically important signaling molecule in various tissues and processes including pain and inflammation [6,7]. Its putative role as a neurotransmitter is supported by recent reports on its effects on hippocampal neurons as well as capsaicin-sensitive peripheral sensory neurons [8-10]. With respect to the latter, there is evidence that intraplantar injection of NaHS (a commonly used H_2_S donor) in rat hindpaws produces mechanical hyperalgesia through activation of T-type Ca^2+ ^channels [11], supporting a pro-nociceptive role for H_2_S. Further, H_2_S generation is also enhanced in the formalin [12] and carrageenan [13] model of persistent inflammatory pain. Systemic injections of H_2_S donors in rats suppress responses to colorectal distention (CRD) by activating K_ATP _channels [14,15], suggesting a possible anti-nociceptive effect. On the other hand, colonic administration of H_2_S enhances pain behaviors in response to CRD in mice [11]. However, the role of H_2_S in non-inflammatory visceral hypersensitivity is not known. Our aim was therefore to study the potential role of H_2_S in the pathogenesis of chronic visceral hyperalgesia (CVH) in a well characterized rat model of irritable bowel syndrome (IBS), developed in our laboratory [16,17]. Our results show that CVH in this model is associated with an upregulation of CBS expression in both thoracolumbar (TL) and lumbarsacral (LS) DRG and that the use of a CBS inhibitor attenuates colonic hypersensitivity. H_2_S enhances the excitability of colon specific sensory neurons *in vitro *and together these findings indicate an important role for H_2_S signaling in IBS-like visceral hyperalgesia. Parts of this work have been published previously in an abstract form [18].

## Methods

### Induction of CVH

Experiments were performed on male Sprague-Dawley rats. Care and handling of these animals were approved by the Institutional Animal Care and Use Committee at the University of Texas Medical Branch and were in accordance with the guidelines of the International Association for the Study of Pain. Ten day old male rat pups received an infusion of 0.2 ml of 0.5% acetic acid (AA) solution in saline into the colon 2 cm from the anus. Controls received an equal volume of saline [16,17]. All experiments were performed at age of 8–10 weeks.

### Cell labeling

Colon specific DRG neurons were labeled by injection of 1,1'-dioleyl-3,3,3',3-tetramethylindocarbocyanine methanesulfonate (DiI, Invitrogen) into the colon wall as described previously [17]. In brief, when the rats were 8 weeks old, animals were anaesthetized with Ketamine (80 mg/kg, i. p.) plus Xylazine (5–10 mg/kg, i. p.). The abdomen was opened by midline laparotomy and the colon was exposed. DiI, 25 mg in 0.5 ml methanol, was injected in ~1 μl volume at 10–15 sites on the exposed colon extending from the level of the bladder to about 6 cm in an oral direction. To prevent leakage and possible contamination of adjacent organs with the dye, the needle was left in place for 1 minute and each injection site was washed with normal saline following each injection. The colon was gently swabbed prior to closing of the abdomen. Animals were returned to their housing and given free access to drinking water and standard food pellets.

### Immunofluorescence study

One-two weeks after DiI injection, rats were perfused transcardially with 150 mL phosphate-buffered saline (PBS) followed by 400 mL ice-cold 4% paraformaldehyde (PFA) in PBS. DRG T_13 _to S_2 _were removed and postfixed for 1 hour in PFA and cryoprotected overnight in 20% sucrose in PBS. Ten-micrometer sections on plus slides were incubated sequentially with CBS or CSE antibody (1:200, Abnova, CA) and then with anti-rabbit Alexa Fluor 488 (1:200, Invitrogen, CA). For triple labeling, sections were simultaneously incubated with TRPV1 (1:200, Santa Cruz, CA) [19] or P2X3R (1:200, Neuromics, MN) [17] antibody and then incubated with Alexa Fluor 355. Negative control was performed by omitting the primary antibody. Sections were viewed with filter cubes appropriate for DiI (rhodamine filter), Alexa 488 and 355. Images were captured and analyzed using Metaview software (Nikon, Melville, NY). To ensure that a neuron was counted only once, serial sections were placed on consecutive slides with at least 50 μm between sections on the same slide.

### Western blotting

Protein extracts from pooled DRGs (T_13_-L_2 _and L_6_-S_2_) and rat colon (divided into three segments: proximal, middle and distal) were prepared in SDS buffer: 50 mM Tris-HCl, 133 mM NaCl, 2%SDS, 1 mM DTT, 1 mM PMSF, 1:100 dilution of protease inhibitor cocktail (sigma), pH = 8. Twenty-five micrograms (25 μg) of protein were fractionated on 10% polyacrylamide gels (Bio-Rad). Proteins were transferred to PDVF membranes (Bio-Rad) at 25 V overnight at 4°C. Membranes was blocked 2 hrs in TBS (50 mM Tris-HCl, 133 mM NaCl, pH = 7.4) and 5% dilution of carnation nonfat milk powder. Primary antibody (anti-CBS or CSE at 1:1000) was incubated for 2 hrs in TBS and 1% milk at room temperature. After washing in TBST (0.5% Tween-20), membranes were incubated with HRP conjugated secondary antibodies (1:5000, Santa Cruz, CA) in TBS and 1% milk for 1 hr at room temperature. Bands were visualized using ECL (Amersham) and exposed to Kodak X-ray film. Membranes were subsequently stripped and re-probed for actin (1:5000, Chemicon, CA) or GAPDH (1:5000, Santa Cruz Biotechnology, CA). Films were scanned and band intensities measured using Optic Quant software (Packard instrument). CBS or CSE data were expressed normalized to actin or GAPDH.

### Behavioral testing for nocifensive responses

Visceral hypersensitivity was measured 8 weeks after neonatal AA treatment by grading the response of rats to colorectal distention (CRD) as described previously [16,17,20]. Briefly, under mild sedation 1% Brevital (25 mg/kg i.p.), a flexible balloon (5 cm) constructed from a surgical glove finger attached to a tygon tubing was inserted 8 cm into the descending colon and rectum via the anus and held in place by taping the tubing to the tail. Rats were placed in small Lucite cubicles and allowed to adapt for 30 minutes. CRD was performed by rapidly inflating the balloon to a constant pressure measured using a sphygmomanometer connected to a pressure transducer. The balloon was inflated to various pressures: 20, 40, 60 and 80 mmHg, for a 20 seconds stimulation period followed by a 2 min rest. Behavioral responses to CRD were measured by visual observation of the abdominal withdrawal reflex (AWR) by a blinded observer and the assignments of an AWR score were as follows: 1 = Normal behavior without response; 2 = Contraction of abdominal muscles; 3 = Lifting of abdominal wall; 4 = Body arching and lifting of pelvic structures.

### Whole-cell patch clamp recordings

As described previously [17], DRGs (T_13_-L_2 _and L_6_-S_2_, bilateral) were dissected out and incubated in dissecting solution with enzymes (collagenase D, 1.5–1.8 mg/ml and trypsin, 1.2 mg/ml; Sigma) for 1.5 hr at 34.5°C. DRGs were then taken from the enzyme solution, washed, and transferred to 2 ml of the dissecting solution containing DNase (0.5 mg/ml). Single cell suspension was subsequently obtained by repeat trituration through flame-polished glass pipettes. Single cell activities were sampled at 100 μs per point and filtered at 2–5 KHz. Resting and action potentials were recorded in external solution, containing (mM): NaCl 130, KCl 5, KH_2_PO4 2, CaCl_2 _2.5, MgCl_2 _1, HEPES 10, glucose 10 (pH = 7.2, adjusted with NaOH, osmolarity = 295–300 mOsm). Unless indicated, patch-clamp pipettes had a resistance of 3–5 MΩ when filled with the pipette solution containing (mM): potassium gluconate 140, NaCl 10, HEPES 10, glucose 10, BAPTA 5 and CaCl_2 _1 (pH = 7.25 adjusted with KOH; osmolarity = 292 mOsm). Action potentials (APs) of colon specific LS DRG neurons were evoked by two times rheobase current stimulation (pulse width: 300 ms) under current clamp conditions.

### Application of drugs

NaHS and HA (Sigma, St Louis, MO, USA) were freshly prepared in normal saline. For behavioral experiment, hydroxylamine (HA, 25 μmol/kg body weight in 1 ml) were intraperitoneally injected 30 min before measurement of the number of nocifensive behavioral response to CRD. For patch clamp experiments, NaHS (250 μM) was applied directly to the recorded cell by pressure and therefore could reach equilibrium almost instantaneously. The switching of solutions was accomplished by two computer-controlled solenoid valves. The solution change rate could be accomplished within 10–15 ms [21].

### Data analysis

Data are expressed as mean ± SEM. Statistical significance for parametric data was analyzed by the Student's *t*-test for comparison between 2 groups and by analysis of variance followed by Tukey's test for multiple comparisons. For AWR behavioral grades, we used a Friedman analysis of variance (ANOVA) to determine whether scores changed with pressure within each experimental group. Median AWR scores at each distention pressure were compared between treatment groups by a Mann-Whitney rank sum test. *P *values less than 0.05 were considered statistically significant.

## Results

### The H_2_S-producing enzyme CBS is expressed by colonic specific DRG neurons

**Figure 1:**
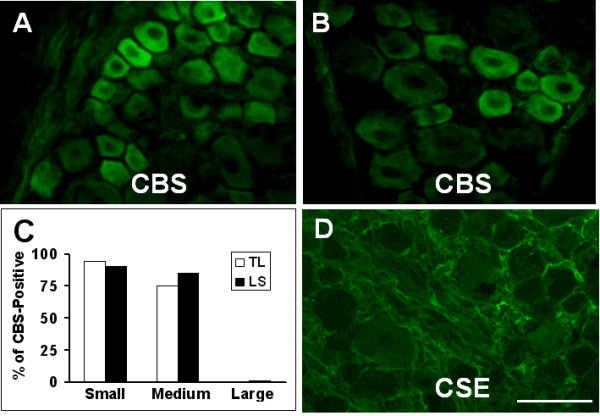
**CBS expression in DRG neurons**. CBS-immunoreactivity (-ir) was observed in L1 (A) and S1 (B) DRG cells from control rat. Note that most of the small and medium sized cells were CBS-ir positive and large cells were weakly labeled or negative. Bar graph showed the CBS-ir in TL and LS DRG neurons (C). CSE-ir was not detected in L1 DRG neurons (D). Bar = 50 μm.

**Figure 2:**
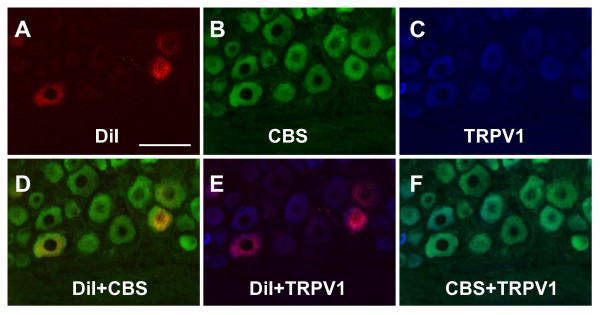
**Co-expression of CBS with TRPV1 in colon specific DRG neurons**. (A) Colon L1 DRG cells were labeled with DiI (red). (B)CBS positive cells were shown in green. (C) TRPV1 positive cells are shown in blue. (D) Merge of double labeling of DiI and CBS. (E) Merge of TRPV1-positive staining and DiI labeling. (F) Merge of TRPV1-positive staining and CBS labeling. Bar = 50 μm.

**Figure 3:**
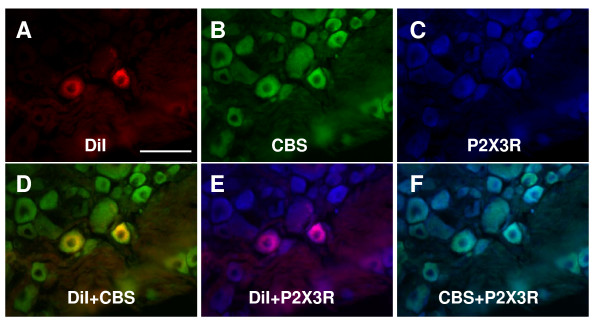
**Co-expression of CBS with P2X3R in colon specific DRG neurons**. (A) Colon L1 DRG cells were labeled with DiI (red). (B) CBS positive cells were shown in green. (C) P2X3R positive cells are shown in blue. (D) Merge of double labeling of DiI and CBS (D). (E) Merge of P2X3R-positive staining and DiI labeling. (F) Merge of P2X3R-positive staining and CBS labeling. Bar = 50 μm.

We examined CBS expression in primary sensory neurons by immunohistochemistry. In rat TL (Fig. 1A) and LS (Fig. 1B) DRGs, CBS was, on average, present in 85.4 ± 3.2% of afferent neurons. CBS was predominantly expressed in small and medium-size neurons, with large DRG neurons staining weakly or not at all (Fig. 1C). The expression of the other H_2_S producing enzyme CSE was also determined. In contrast to the finding in CBS, the CSE antibodies did not reveal reliable and reproducible staining in rat DRG neurons and quantitative assessment was therefore not feasible (Fig. 1D). To determine whether colon specific DRG neurons expressed the CBS, double labeling studies were performed. Colon specific DRG neurons were retrogradely labeled by DiI (Fig. 2A&3A) which was injected into the colon wall 7–10 days before euthanasia. CBS-immunoreactivity (Fig. 2B&3B) was present in all DiI labeled colon specific DRG neurons (Fig. 2D&3D).

### CBS is co-localized with TRPV1 and P2X3R in colon specific DRG neurons

We next examined whether CBS was co-expressed in TRPV1- or P2X3R-positive colon specific DRG neurons since these are two major molecules involved in transduction of nociceptive information. Triple-labeling techniques were used in this experiment. Colon specific DRG neurons were retrogradely labeled by DiI, as above. DRG sections containing DiI labeled neurons were stained with CBS and P2X3R or CBS and TRPV1 antibodies. All colon specific DRG neurons that were immunoreactive for CBS also were positive for TRPV1 (Fig. 2C, E&2F). Similarly, all colon specific DRG neurons that were immunoreactive for CBS also were positive for P2X3R (Fig. 3C, E&3F).

### CBS expression is upregulated in the DRGs and colon in a rat model of CVH

**Figure 4:**
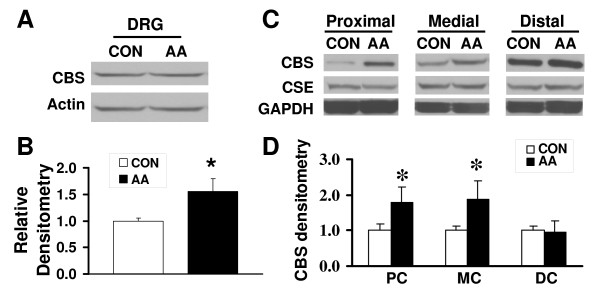
**Upregulation of CBS expression in DRGs and the colon**. (A, B) Upregulation of CBS expression in colon related DRGs from neonatal AA treated rats. (C, D) CBS expression was increased in proximal and medium colon in neonatal AA-treated rats. Actin or GAPDH was used as a loading control for DRGs and colon, respectively. CSE expression was not significantly altered in colons from rats with neonatal AA treatment (C). n = 4 for each group. *p < 0.05 compared with controls.

We then determined the effect of neonatal colonic infusion of acetic acid (AA) on CBS expression in DRG and colon. Ten-day old pups received a single colonic infusion of 0.2 ml of AA. Eight weeks later, the expression of CBS in colon-related DRGs (T_13_-L_2 _and L_6_-S_2_, bilateral), as assessed by Western blotting of protein extracts, was significantly greater than that from control rats (Fig. 4A&4B, p < 0.05). In addition, a significant increase of CBS expression was noted in proximal and medium colon (but not distal colon) from rats with neonatal AA injection (Fig. 4C&4D). We also examined the expression of CSE in rat colon. As shown in figure 4C, CSE expression in the colon was not significantly altered 8 weeks after neonatal AA treatment.

### CBS inhibitor attenuates abdominal withdrawal reflex (AWR) scores in a rat model of CVH

**Figure 5:**
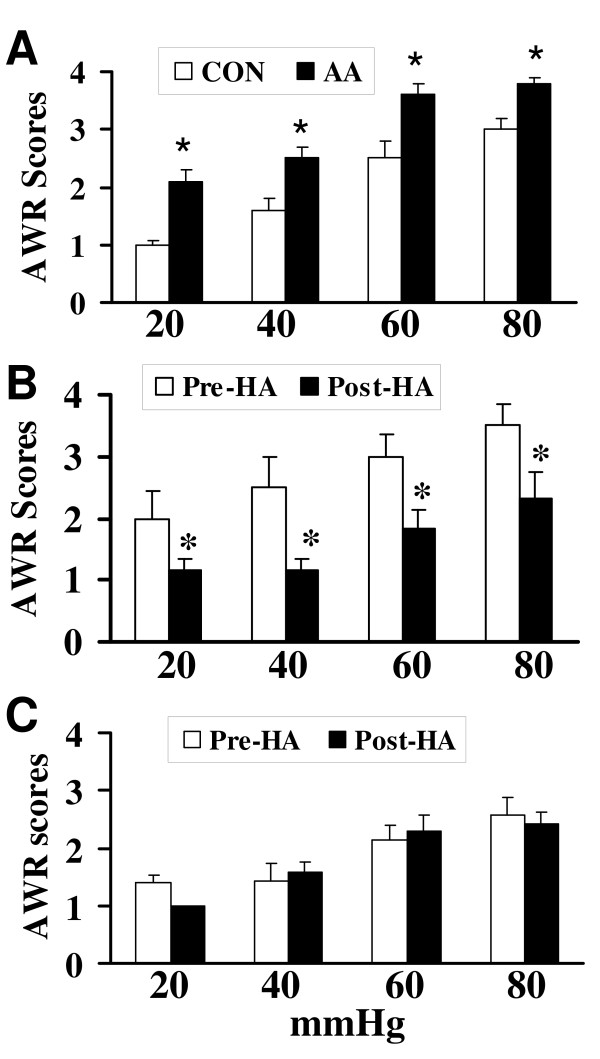
**Effect of CBS inhibitor on AWR (abdominal withdrawal reflex) in response to colorectal distention (CRD)**. (A) Neonatal AA-treatment significantly enhanced the AWR scores. *p < 0.05 compared with controls. (B) CBS inhibitor hydroxylamine (HA, 25 μmol/Kg body weight) significantly attenuated the AWR scores in neonatal AA-treated rats. *p < 0.05 compared with Pre-HA. (C) HA did not produce any effect on AWR scores in healthy control rats.

Visceral sensitivity was determined by measuring the AWR scores in response to colorectal distention (CRD) at 8–10 weeks of age. In accordance with our previously reported results [16,17], the AWR scores were significantly higher in neonatal AA-treated rats at 20, 40, 60 and 80 mmHg distention pressures than those in saline treated rats (Fig. 5A, *P < 0.05). We then examined the change in response to CRD after administration of the CBS inhibitor, hydroxylamine (HA), 30 minutes before balloon distention. We used a relatively high concentration of this agent (25 μmol/kg in 1 ml), a dose similar to those selected by other investigators [22]. In sensitized rats, HA treatment caused a significant decline in the mean AWR scores to CRD at 20, 40, 60 and 80 mmHg when compared with pre-injection (Fig. 5B). In control rats, HA had no significant effects on the AWR scores (Fig. 5C), suggesting that this agent did not act as a non-specific analgesic and that CBS do not normally participate in the responses to CRD.

### The H_2_S donor NaHS increases excitability of colon specific DRG neurons

**Figure 6:**
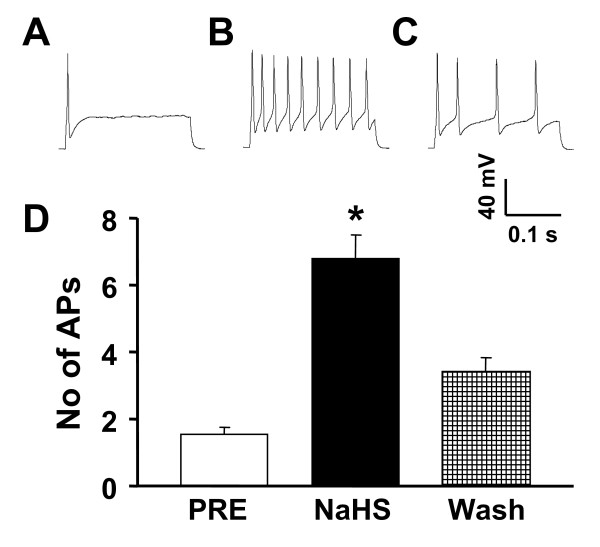
**Alteration in action potential (AP) frequency**. Representative traces of APs were induced by 300-ms depolarizing current pulses injected through the patch pipette at two times rheobase in DiI labeled neurons from control rats under current-clamp conditions. (A) AP recorded before (PRE) application of H_2_S donor NaHS. (B) APs recorded after 2 mins perfusion of H_2_S donor NaHS (250 μM). Application of NaHS significantly increased the APs frequency evoked by two times rheobase electrical stimulation. (C) APs recorded after 10 min wash. (D) Bar graph showing a significant increase in average number of APs elicited by a two times rheobase current injection after application of NaHS (*P < 0.01 compared with PRE).

Since our previous experiments demonstrated that neonatal AA treatment increased colon specific DRG neuronal excitability [23], we determined whether H_2_S mimicked the effects induced by neonatal AA treatment. Perfusion of freshly prepared H_2_S donor NaHS (250 μM) for 2 mins in LS DRG (L6-S2, bilateral) neurons from control rats (n = 7) did not alter the resting membrane potential (RP) in the majority of recorded cells (22/33, 66.7%). Only 6 cells showed a slight depolarization and 5 cells displayed a slight hyperpolarization (data not shown). This is in consistent with our previous findings that neonatal AA treatment did not alter RPs of colon specific LS DRG neurons [23]. However, perfusion of NaHS led to a significant increase in numbers of action potentials (APs) in 68.2% of the neurons (n = 15) evoked by two times rheobase current stimulation (Fig. 6B&6D). The remaining 7 cells did not show any changes in numbers of APs evoked by two times rheobase current stimulation. The average of numbers of APs for all 15 neurons after NaHS application were 6.8 ± 0.7, which is significantly higher than those before NaHS application (PRE 1.5 ± 0.2, n = 15, *p < 0.05). This effect lasted about 10 minutes after wash (Fig. 6C), indicating that H_2_S has a long term effect on excitability of colon specific DRG neurons. These data demonstrated that H_2_S donor NaHS enhanced excitability of colon specific DRG neurons.

## Discussion

Our study shows that the H_2_S producing enzyme cystathionine β-synthase (CBS) is expressed by a subpopulation of primary sensory neurons (Figs. 1, 2 &3) and is upregulated in a rat model of IBS-like chronic visceral hyperalgesia (Fig. 4). CBS upregulation may contribute to chronic visceral hyperalgesia since a CBS inhibitor significantly attenuates the AWR scores in neonatal AA-treated rats (Fig. 5). In addition, H_2_S donor NaHS greatly enhanced the frequency of action potentials of DRG neurons *in vitro *(Fig. 6). These data strongly suggest that CBS-H_2_S signaling may play an important role in "functional" visceral pain i.e. pain occurring in the absence of overt structural or inflammatory processes.

There is considerable support for a role of H_2_S as a neuromodulator [24-26] or an endogenous gaseous transmitter [27]. In physiological conditions, H_2_S has been found to regulate key neuronal functions, including induction of long-term potentiation and modulation of NMDA receptor currents in the hippocampus [24,28]. H_2_S has been reported to produce inward or outward currents on dorsal raphe serotonergic neurons *in vitro *[29]. H_2_S can also regulate the release of corticotrophin-releasing hormone from the hypothalamus [30]. H_2_S is an important endogenous vasoactive factor and is an identified gaseous opener of K_ATP _channels in vascular smooth muscle cells [27].

Endogenous H_2_S is also an important mediator of inflammation in a variety of models [6,31]. Along with this, there is growing evidence of its involvement in nociception in both somatic [32,33] and visceral [11,14,15] organs. However, this role is likely to be complex as suggested by the somewhat conflicting reports in the literature. Distrutti and colleagues have shown that systemic administration of H_2_S donors attenuates the response to CRD in both healthy and post-colitic rats; this effect is sensitive to glibenclamide, suggesting that it is mediated by K_ATP_channels [14,15]. On the other hand, intracolonic H_2_S donor NaHS enhanced spontaneous visceral pain behavior as well as referred hyperalgesia and spinal ERK expression in mice, an effect that appears to be mediated by T-type calcium channels as it is blocked by mibefradil but not by verapamil (an L-type channel blocker) or glibenclamide [11]. The reasons for these discrepant findings may include but not limit to H_2_S concentration, effect of inflammation on H_2_S action and H_2_S action sites. The concentration of H_2_S may not be an explanation for the different results since the same dose of H_2_S used by two different groups produced the different effects [11,14,15]. Tissue inflammation may have an influence on H_2_S actions. In this animal model, however, no histological signs of inflammation/injuries or significant changes in MPO activities were observed in the colons 8 weeks after neonatal AA treatment as reported previously [16]. Thus, the different effects of H_2_S in AA-treated rats were not due to inflammation/injury. The site of action or/and the source of H_2_S may be most likely related to the different effect of H_2_S as has been suggested in somatic pain models [33]. Thus, systemic administration of exogenous H_2_S may activate central antinociceptive mechanisms whereas peripheral H_2_S administration or endogenous sources may invoke pro-nociceptive effects. Further experiments on the mechanism of H_2_S signaling pathway are warranted.

CBS and systathionine γ-lyase (CSE) are two important enzymes for generation of endogenous H_2_S [1-5]. These two enzymes have been found in many types of mammalian cells in the central nervous system as well as peripheral tissues [10,25,27,34,35]. Both enzymes have also been shown to be expressed by rat colonic tissue [15]. CSE and CBS have also been localized to colonic enteric neurons and CSE, but not CBS, to interstitial cells of Cajal in guinea pig colon [10]. We have confirmed previous studies on the expression of CBS and CSE in the colon and further have shown that CBS, but not CSE, is expressed by colon-specific sensory neurons (Fig. 1), where it is localized to nociceptive neurons, indicating that CBS is a major enzyme responsible for the endogenous production of H_2_S in these cells. We have also shown that CBS expression in both sensory neurons and the colon is dynamic and is upregulated in a model of chronic non-inflammatory visceral hypersensitivity. Theoretically, enhanced H_2_S production from either a colonic or a neuronal source can affect the function of sensory neurons in our model and contribute to both enhanced pain as well as the secretomotor response that has previously been shown in guinea pigs [10]. An additional source of H_2_S comes from sulfate-reducing bacteria in the GI tract [36-38]. Further research will indicate the relative importance of these various sources in health and disease. Our studies showed that CBS inhibitor attenuated the AWR scores in neonatal AA-treated rats (Fig. 5B) and no significant effect was seen in control rats (Fig. 5C), suggesting that this was not a non-specific analgesic effect. This also suggests that the role of CBS in signaling colonic distension may not be as important in health as in the sensitized state. Taken together, our results suggest but do not prove that CBS may be an important source of endogenous H_2_S and a credible therapeutic target for visceral pain syndromes.

Although the detailed mechanisms by which H_2_S induces visceral hyperalgesia have yet to be fully investigated, our data and that of others suggest that colonic nociceptors are a prime site of action. H_2_S has been shown to enhance the excitability of enteric neurons, possibly via TRPV1 receptors on extrinsic afferent terminals [10]. Others have shown that stimulation with H_2_S enhances T-type calcium currents in small sensory neurons *in vitro *[11]. In this study, we provide new evidence for the first time to show that H_2_S donor NaHS increased the number of action potentials evoked by electrical stimulation in colon specific DRG neurons (Fig. 6), indicating that H_2_S may increase the neuronal excitability. This effect may result from the previously reported activation of T-type calcium channels and/or involve potassium and/or sodium ion channels. In addition, we showed that CBS-ir was present abundantly in small and medium-size neurons of DRGs and they co-localized with TRPV1 and P2X3 receptors, suggesting a possible interaction between these molecules. Both of these receptors are upregulated in our model of IBS-like pain [16,17] and further studies are needed to investigate whether and how H_2_S modulates their function and/or expression.

In conclusion, although there is a discrepancy of H_2_S effects in the literature, our data demonstrate that CBS-H_2_S signaling pathways may play a role in chronic visceral hyperalgesia, even in the absence of overt inflammation of the colon wall. Our results also identify CBS as a potential target for novel agents for the treatment of visceral pain in IBS and related disorders.

## Competing interests

The authors declare that they have no competing interests.

## Authors' contributions

All authors have read and approved the final manuscript. GYX designed, performed and supervised the experiments, analyzed the data, prepared the figures and wrote the manuscript; MS and SFZ performed the experiments; JHW, JDZC and PJP coordinated the project, helped to interpret the data, and edited the manuscript.
